# Experiencing and designing community-based medicine – development and evaluation of an elective based on explorative learning 

**DOI:** 10.3205/zma001282

**Published:** 2019-11-15

**Authors:** Wolfram J. Herrmann, Sabine Gehrke-Beck, Christoph Heintze

**Affiliations:** 1 FH Münster, Fachbereich Gesundheit, Münster, Germany; 2 Charité – Universitätsmedizin Berlin, Institut für Allgemeinmedizin, Berlin, Germany

**Keywords:** health education, public health, primary health care, community medicine, teaching, explorative learning

## Abstract

**Objective: **To develop and evaluate an elective for the 6^th^ semester in the medical curriculum at Charité – Universitätsmedizin Berlin. In this elective, medical students could experience and test Community Oriented Primary Care, hence the integration of public health into primary care, by using explorative learning methods.

**Method: **In three consecutive semester, all participants of the elective filled in a questionnaire before and after the elective. The self-developed questionnaire covered socio-demographic features, an evaluation of the elective as well as a self-assessment regarding learning objectives and attitudes. The results were analyzed descriptively; the learning success was measured by mixed model regression.

**Results: **Thirty-one students (100% of the elective participants) took part in the evaluation, 30 of them (96.8%) at both survey dates. The students evaluated the elective and particularly the commitment of the teachers as very positive. The five-level Likert scale showed a significant growth of knowledge by an average of 1.3 points. The attitudes of the students hardly changed.

**Conclusion:** Students can experience Public Health practically by means of Community Oriented Primary Care. In doing so, explorative learning is an appropriate method providing a significant increase in competences.

## Zusammenfassung

**Zielsetzung: **Ziel war es, ein Wahlpflichtfach für das sechste Semester im Modellstudiengang Medizin an der Charité – Universitätsmedizin Berlin zu entwickeln und zu evaluieren. In diesem Wahlpflichtfach können Studierende mittels forschenden Lernens Community Oriented Primary Care, also die Integration von Public Health in die hausärztliche Versorgung, kennenlernen und erproben.

**Methodik:** In drei konsekutiven Semestern (WS 2016/2017– SS 2018) wurden alle Teilnehmenden des Wahlpflichtfaches zu Beginn und nach Abschluss des Wahlpflichtfaches mittels eines selbstentwickelten Fragebogens befragt. Es wurden soziodemographische Merkmale, die Beurteilung des Wahlpflichtfaches sowie eine Selbsteinschätzung hinsichtlich der Lernziele und thematisch relevanter Einstellungen abgefragt. Die Analyse erfolgte deskriptiv und hinsichtlich des Lernerfolges als Mixed Modell Regression.

**Ergebnisse: **Einunddreißig Studierende (100% der Teilnehmer am Wahlpflichtfach) nahmen an der Evaluation teil, davon 30 (96,8%) zu beiden Befragungszeitpunkten. Die Studierenden bewerteten das Wahlpflichtfach insgesamt und insbesondere das Engagement der Dozierenden sehr positiv. Hinsichtlich der Lernziele ergab sich ein deutlicher Wissenszuwachs von durchschnittlich 1,3 Punkten auf einer fünfstufigen Likertskala. Die Einstellungen der Studierenden änderten sich kaum.

**Schlussfolgerung:** Public Health kann mittels Community Oriented Primary Care praktisch vermittelt werden. Die Methode des Forschenden Lernens ist gut geeignet und führt zu einem deutlichen Kompetenzgewinn.

## 1. Background

Social conditions have an important impact on health, illness and life expectancy. Even within Berlin, life expectancy differs depending on the place of residence or social status [1], [2]. Community Oriented Primary Care (COPC) considers these aspects in medical practice by integrating public health into GP care [3], [4]. COPC is oriented towards medical approaches [5]: In COPC, anamnesis and examination, diagnosis, treatment plan and evaluation of successful treatment are transferred to a community or neighbourhood: In a first step, on-site data are collected thus providing a community diagnosis. This community diagnosis enables to plan and implement an intervention. Subsequently, the success of the intervention has to be evaluated. Due to this desired parallelism to medical action, Greenhalgh [6] considers Community Oriented Primary Care “a biomedical theory adapted to a community development“. For Greenhalgh and the European Regional Branch of the World Organization of Family Doctors (WONCA Europe) community orientation is an integral part of general practice [6], [7].

Even though medical studies in Germany, Austria and Switzerland cover social aspects of health and illness on a theoretical basis, action-oriented approaches are still lacking [cf. e.g. [8]]. Up to now, Community Oriented Primary Care has not yet played a role in medical studies. In other countries, however, appropriate teaching concepts have already been implemented, e.g. in Belgium at Ghent University. There, all students have to work in a deprived community for a week in order to make a community diagnosis [9]. 

## 2. Objective

It was our aim to develop, test and evaluate an elective on Community Oriented Primary Care for students in the 6^th^ semester in the medical curriculum of Charité – Universitätsmedizin Berlin [cf. [10]]. 

## 3. Concept

### 3.1. Didactic concept

This elective uses explorative learning (research-based learning) as didactic concept [11], [12]. Core aspect of this method is the active research of the students enabling them to learn during the research process. Another important aspect is that the students have to reflect their research activity. The unpredictability of this process is a challenge for the lecturers: Which topics do the students choose? Which methods do they use? Therefore, the concept demands a high degree of flexibility from the lecturers.

Unlike in other concepts such as those of Pfadenhauer et al. [13], explorative learning as used here does not primarily aim at increasing research competences, but is a method for the general transfer of knowledge and competence, which should be used in this elective subject to convey learning content and competences from the field of public health.

#### 3.2. Learning objectives

##### 3.2.1. Primary learning objective

Students can survey the social and regional influences on health and illness in a neighbourhood and use them for their medical practice.

##### 3.2.2. Specific learning objectives

- Students understand that the physical and social environment of patients is related to their health and well-being.
- Students can discuss the influence of a neighbourhood on the health of the population.
- Students can discuss the influence of a neighbourhood on the practice of family doctors.
- Students can name exemplary basic epidemiological methods of health monitoring.
- Students can describe Community Oriented Primary Care including its core concepts and processes.
- Students can characterize a certain population based on data.
- Students can determine a health problem at community level based on subjective and/or objective data.
- Students can illustrate the diversity of health and disease-related services using a neighbourhood as an example.

#### 3.3. Course of the elective

The compulsory elective was organized as a block training (until the 2017 spring semester with a duration of four weeks, from the 2017/2018 fall semester with a duration of three weeks). In each semester, the students explored a different neighbourhood (local planning region).

At the beginning, the students got to know each other. They introduced the neighbourhood in which they lived to the other students and explained what they could contribute to the elective. They learned the theoretical concepts of Primary Care and Community Oriented Primary Care and the methodological foundations of participant observation. After that, the students went in small groups and alone for two days to the neighbourhood and explored it by participant observation. Then all students came together, reported about their experiences and which health-relevant problems they had encountered. The students agreed on two to four of these problems, which they wanted to elaborate on in small groups. In order to be able to conduct independent research, the students now received an introduction to the basics of health care research. In small groups, they planned their methodical approach and carried it out. Mentors from the institute supervised the students. At the end of the compulsory elective, the students presented the results to local actors such as the responsible district councilor.

#### 3.4. Examination format

The examination took place in a formative way. The examination format was a portfolio, in which the students documented their work in the elective. For the portfolio, the students were given a rough structure for their orientation. This portfolio structure consisted of three parts:

- Short presentation of the neighbourhood in texts, photos, etc.
- Final presentation of their results
- Reflection of their own approach.

The portfolio rated as passed, when it met following criteria:

- The work in the elective was presented in a verifiable way
- The work in the elective was presented comprehensively, including all essential worksteps as well as the description of a health problem in a neighbourhood
- The work in the elective was reflected self-critically

## 4. Evaluation method

In the fall semester 2016/2017, spring semester 2017 and fall semester 2017/2018, the elective was evaluated. For the quantitative scientific evaluation of the compulsory elective, the students received an anonymous questionnaire survey on the first day at the beginning of the compulsory elective subject and on the last day. A pseudonymous code was used to match the questionnaires of the pre- and post-surveys. The questionnaire contained questions on attitudes, self-assessment with regard to learning goals and questions on the evaluation of the elective. Table 1 (Tab. 1) shows the self-assessment questions broken down by learning objectives and attitudes. In order to ensure the anonymity of the evaluation, sociodemographic data were collected in a separate non-matched questionnaire. 

A descriptive evaluation was conducted. In order to assess the learning success, a mixed model regression with the mean values of the self-assessment regarding the learning objectives as a dependent variable was carried out. The students (with values before/after) and the three cohorts were two levels in the regression. The independent variable was the dummy variable, whether it was the pre- or post-survey. Other variables were not included in the regression analysis. The analysis was performed using the statistics program R; the lme4 package was used for mixed model regression.

## 5. Results of the evaluation

In the fall semester 2016/2017 eleven students, in the spring semester 2017 eight students and in the fall semester 2017/2018 twelve students took part in the elective (n=31). The compulsory elective was open to about 330 students, with a maximum of 16 participants. The response to the socio-demographic questionnaire and the questionnaire at the beginning of the compulsory elective was 30 (97%) and the response to the questionnaire at the end of the compulsory elective was 100%.

The majority of the students was female (70%); the age varied from 20 to 36 years with a mean of 24.5 (SD=4.1) years. 13.3% of the students had children of their own. 

**Figure 1:**
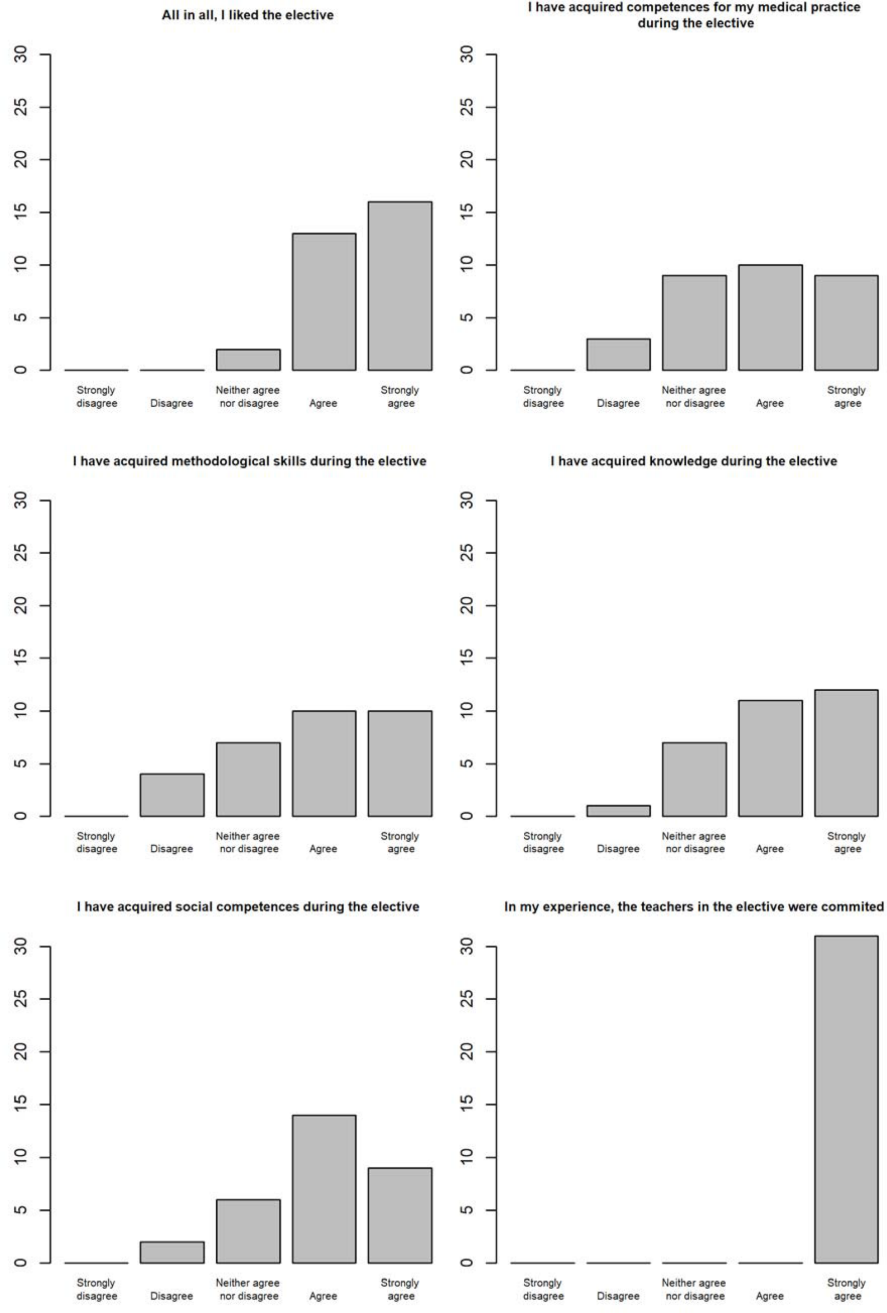
Evaluation of the elective at the end (n=31)

After the elective, the students rated the study offer as very good (see figure 1 (Fig. 1)): all students rated the commitment of the lecturers as very high. The students stated that they had attained social and methodological competences as well as knowledge of the contents and competences for their medical practice.

For the self-assessment of the students, we retrieved the learning goals and attitudes before and after the elective. Table 1 (Tab. 1) shows the absolute changes in this self-assessment of the elective subject. There was a clear improvement in the learning objectives of an average of 1.26 (SD=0.45) points compared with hardly any change in the attitudes of 0.29 (SD=0.19) points in the intended direction. 

**Figure 2:**
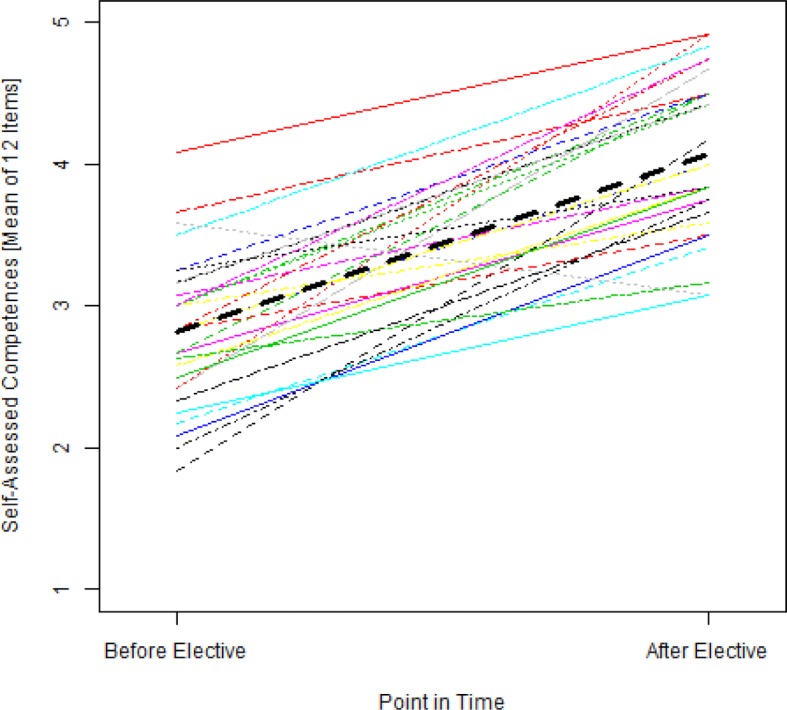
Spaghettiplot of the self-assessed competences of the individual students before and after the elective with a super imposed regression line (higher values mean higher self-assessed competence, n=30)

In mixed model regression, the result is an intercept of 2.82 (SE=0.10) with an effect of 1.25 (SE=0.11) points. The random effect for the intercept of the participants has a variance of 0.11; for the cohorts, the variance of the random effect of the section is 0.00 and the slope <0.01. This means that regardless of the cohort, the students increased their self-assessment learning objectives by 1.25 points from an average of 2.82 points. Figure 2 (Fig. 2) shows the spaghetti plot of the individual students with the regression line above it. 

## 6. Discussion

Our results show that a module on Community Oriented Primary Care can be successfully implemented as a compulsory elective in medical studies using methods of explorative learning. The elective “Experiencing and Designing Community-based Medicine” was rated as very positive by the participants. In the self-assessment, a pre-post comparison showed a clear increase in competences with regard to competence-based learning objectives and only a slight effect with regard to changed attitudes.

One limitation to this evaluation is that no comparison group was surveyed. However, we could observe a significantly smaller change in the pre-post comparison with regard to the attitudes surveyed. Since these are also subject to the effects of social desirability and an intervention effect, this indicates that the gain in competences is nevertheless relevant. However, the self-assessment with regard to attitudes was already higher at the beginning of the module, so that ceiling effects can also be expected here. This was favoured by the fact that it was a compulsory elective subject. Therefore, there is a selection bias, i.e. students possibly took part in the course with a more positive attitude in advance. A further limitation is that learning success was measured based on self-assessments. One strength of the evaluation is that all students took part. Only at the beginning of the elective there was one student who did not participate in the survey.

In comparison to the evaluation of Art et al. [9], similar positive feedback from the students results. Unlike with a small part of the students in the evaluation of Art et al, there is no negative attitude in our evaluation. This is possibly because for the students surveyed at Art et al, the module was a compulsory course, whereas our module was an elective course. However, possibly some students were assigned to it, without having chosen it.

## 7. Conclusion

As our results show, practical teaching of public health by means of Community Oriented Primary Care is possible in medical studies. Additionally, our findings reveal that explorative learning based on the methods of social science research fits into the framework of medical studies as an elective. To what extent the concept can become part of the compulsory curriculum, as in Ghent [7], in the German-speaking countries, will have to be tested and further investigated in the future.

During implementation, it became apparent that such a concept is time-consuming and personnel-intensive due to its preparation and monitoring. It requires a high degree of flexibility from the lecturers in terms of content and methodology.

Since the relevance of interdisciplinary and multi-professional training in medical studies is becoming increasingly important, Community Oriented Primary Care approaches in medical studies should be further strengthened with a view to overcoming sectoral boundaries in the health care system. Based on the positive evaluation we can say that the concept “Experiencing and designing Community-based medicine” provides a promosing approach.

## Competing interests

The authors declare that they have no competing interests. 

## Figures and Tables

**Table 1:**
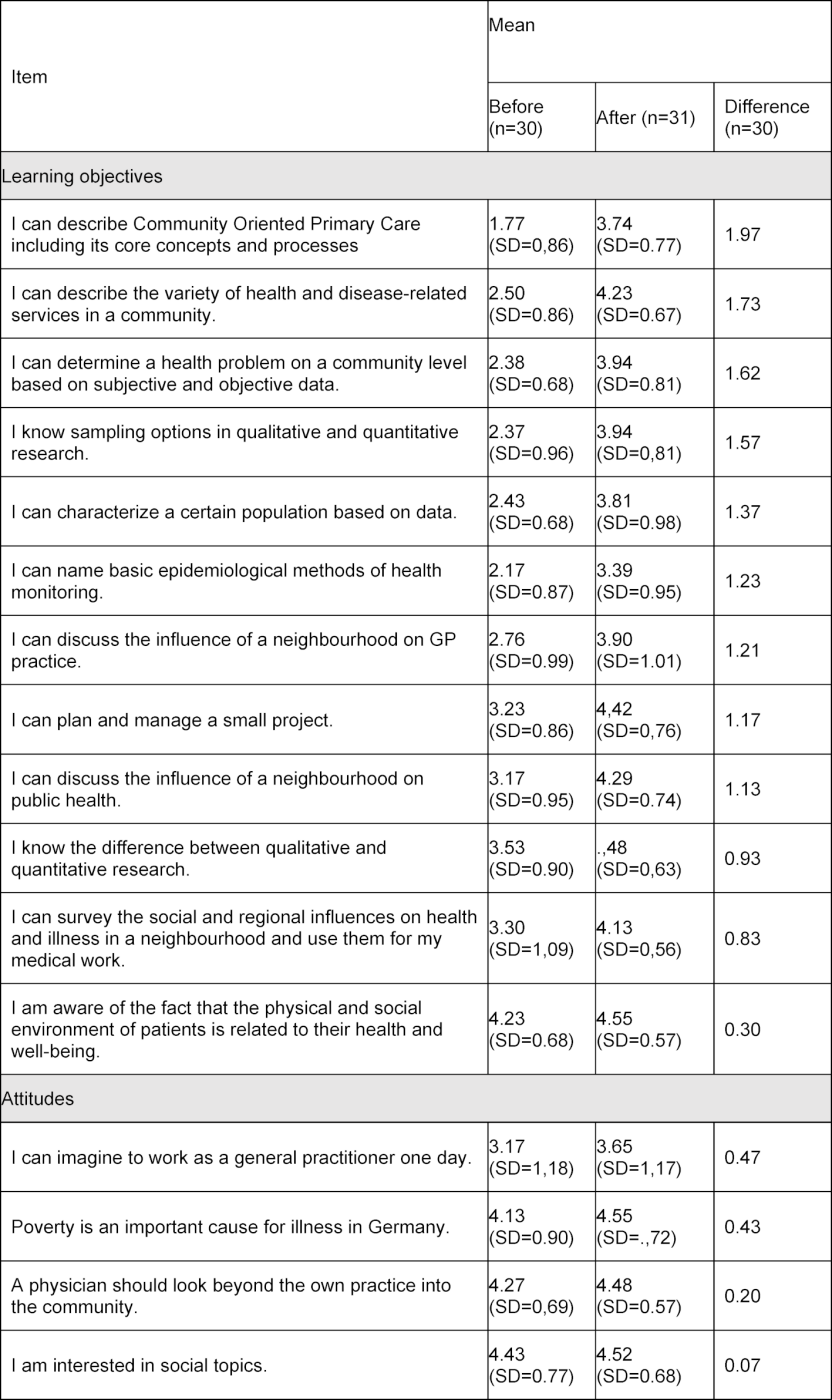
Self-assessment before and after the elective and difference before and after on a five-level Likert scale with classification of the items into learning objectives and attitudes.

## References

[1] Lampert T, Kroll LE (2014). Soziale Unterschiede in der Mortalität und Lebenserwartung. GBE kompakt.

[2] Meinlschmidt G (2014). Handlungsorientierter Sozialstrukturatlas Berlin 2013.

[3] Tollmann S (Soc). Community oriented primary care: origins, evolution, applications.

[4] Iliffe S, Lenihan P (2003). Integrating primary care and public health: learning from the community-oriented primary care model. Int J Health Serv.

[5] Blumenthal D (2009). Clinical community health: revisiting "the community as patient". Educ Health.

[6] Greenhalgh T (2007). Primary health care. Theory and practice.

[7] European Academy of Teachers in General Practice (Network within WONCA Europe) (2011). The European Definition of General Practice/Family Medicine.

[8] Siebers L, Hensen P, Roeder N (2007). Gesundheitsökonomie, Gesundheitssystem und öffentliche Gesundheitspflege im Medizinstudium. Gesundheitsök Qualitätsmanag.

[9] Art B, De Roo L, Willems S, De Maeseneer J (2008). An Interdisciplinary Community Diagnosis Experience in an Undergraduate Medical Curriculum: Development at Ghent University. Acad Med.

[10] Herrmann WJ, Gehrke-Beck S, Heintze C (2017). Kiezmedizin erleben und gestalten. Gesundheit braucht Politik. Z Soz Med.

[11] Bundesassistentenkonferenz (1970). Forschendes Lernen - Wissenschaftliches Prüfen. Schriften der Bundesassistentenkonferenz.

[12] Huber L, Hellmer J, Schneider F (2009). Forschendes Lernen im Studium: aktuelle Konzepte und Erfahrungen.

[13] Pfadenhauer L, Coenen M, Kühlmeyer K, Odukoya D, Schunk M, von Unger H (2018). Teaching Qualitative Research Methods in Public Health and Medicine: a research oriented module. GMS J Med Educ.

